# Community cognitive interviewing to inform local adaptations of an e-mental health intervention in Lebanon

**DOI:** 10.1017/gmh.2018.29

**Published:** 2018-11-27

**Authors:** J. Abi Ramia, M. Harper Shehadeh, W. Kheir, E. Zoghbi, S. Watts, E. Heim, R. El Chammay

**Affiliations:** 1 National Mental Health Programme (NMHP)|Ministry of Public Health, Lebanese University Central Directorate, Museum Square, 9800, Beirut, Lebanon; 2 Faculty of Medicine, The Institute of Global Health, University of Geneva, Geneva, Switzerland; 3 Public Health, Universite Libanaise Faculte des Sciences, Beirut, Lebanon; 4 Medical School, University of Sydney – Sydney Medical School Nepean, Sydney, New South Wales, Australia; 5 Department of Psychology, University of Zurich, Zurich, Switzerland

**Keywords:** Cultural adaptation, E-intervention, E-mental health, interventions, low-resource setting, minimally guided interventions

## Abstract

**Background.:**

Lebanon has a need for innovative approaches to increase access to mental health care to meet the country's current high demand. E-mental health has been included in its national mental health strategy while in parallel the World Health Organization has produced an online intervention called ‘Step-by-Step’ to treat symptoms of depression that is being tested in Lebanon over the coming years.

**Aim.:**

The primary aim of this study is to conduct bottom-up, community-driven qualitative cognitive interviewing from a multi-stakeholder perspective to inform the cultural adaptation of an Internet-delivered mental health intervention based on behavioural activation in Lebanon.

**Methods.:**

National Mental Health Programme staff conducted a total of 11 key informant interviews with three mental health professionals, six front-line workers in primary health care centres (PHCCs) and two community members. Also, eight focus group discussions, one with seven front-line workers and seven others with a total of 66 community members (Lebanese, Syrians and Palestinians) were conducted in several PHCCs to inform the adaptation of Step-by-Step. Results were transcribed and analysed thematically by the project coordinator and two research assistants.

**Results.:**

Feedback generated from the cognitive interviewing mainly revolved around amending the story, illustrations and the delivery methods to ensure relevance and sensitivity to the local context. The results obtained have informed major edits to the content of Step-by-Step and also to the model of provision. Notably, the intervention was made approximately 30% shorter; it includes additional videos of content alongside the originally proposed comic book-style delivery; there is less emphasis on total inactivity as a symptom of low mood and more focus on enjoyable activities to lift mood; the story and ways to contact participants to provide support were updated in line with local gender norms; and many of the suggested or featured activities have been revised in line with suggestions from community members.

**Conclusions.:**

These findings promote and advocate the use of community-driven adaptation of evidence-based psychological interventions. Some of the phenomena recorded mirror findings from other research about barriers to care seeking in the region and so changes made to the intervention should be useful in improving utility and uptake of ‘Step-by-Step’.

## Introduction

E-mental health (i.e. the use of Internet and mobile phones for delivering mental health treatment) has enormous potential to increase access to evidence-based interventions for mental and behavioural disorders, particularly in middle-income countries with wide smartphone use and Internet coverage such as Lebanon (Arjadi *et al*. 2015). Lebanon is a small country with a high mental health care need due to a history of conflict and political unrest, an under-resourced and an influx of almost 1.5 million Syrian refugees (UNHCR, 2018). Estimates from 2006 suggest that just 10% of people in Lebanon who had a mental disorder had access to treatment (Karam *et al*. 2006), a figure that has possibly worsened since the refugee crisis. Smartphone use and Internet coverage in Lebanon are high, with an estimated 96% of people having a mobile phone subscription and 76% of people regularly using the Internet (International Telecommunications Union, 2017). A recent United Nations report estimates that Syrian refugee communities are also known to have high accessibility to smartphones and the Internet, at approximately 80% household usage (UNICEF *et al*. 2017).

The World Health Organization (WHO) has developed an Internet-delivered behavioural activation intervention to treat symptoms of depression among adults, called *Step-by-Step* (Carswell *et al*. 2018). The intervention consists of five approximately 30 min sessions that are delivered on a weekly basis over 8 weeks (to allow flexibility of pace for users). Step-by-Step predominantly uses psychoeducation, behavioural activation (including specifically a session on social support activation) and some simple relaxation techniques. The Step-by-Step sessions consist of a narrated story of a character who has learned how to better manage his/her mood from their doctor and an interactive part where the user can plan his/her own activities for the week to come. This story can be watched via video of a slideshow of still images and a voice over, or read like a comic book where the user swipes to see the next illustration with the text beneath. A story was used as the vehicle to transmit the therapeutic content, as stories and storytelling were deemed to be globally and culturally universal and engaging from an anthropological perspective. The intervention includes audio-recorded breathing and grounding exercises that beneficiaries can listen to. The companion in the story is tailored according to the gender of the client, and users can choose between characters that may resonate with some of the cultural groups in Lebanon (e.g. woman with/without a headscarf or man with/without a beard). It is noteworthy that the story content is common to all genders in terms of symptoms expressed, with slight adaptations in the activities to enhance relevance to each gender. Beneficiaries are asked to apply the exercises and activities between the sessions. The intervention is supported by trained non-specialist assistants (called ‘e-helpers’) who have weekly phone or message-based contact with users to provide support and guidance, lasting around 15 min per week.

The WHO, the Ministry of Public Health (MoPH) in Lebanon and other project partners are currently testing the feasibility of using Step-by-Step in Lebanon in a pilot study. Following this initial pilot, Step-by-Step is being tested in a number of fully powered randomised controlled trials in Germany, Sweden, Egypt and in Lebanon, commencing in 2018. Step-by-Step had been written in a generic manner and with global usage in mind, but in order to use Step-by-Step in Lebanon, it was necessary to sensitively adapt the intervention to the culturally diverse local setting, considering the three main population groups: Lebanese, Palestinians and Syrians.

Lebanon has a varied cultural landscape, owing to rich religious diversity, a complex colonial and migration history and a highly politicised post-conflict environment. A systematic review identified 22 studies on the use of Western-developed interventions in the Middle East. In this review, cultural incompatibility accounted for the majority (54%) of barriers to implementation. Further barriers were lack of public awareness around mental health; gender-related norms; stigma and diminished social status of people with mental health problems; and language and presentation of distress (Gearing *et al*. 2013). This review shows that when delivering psychological interventions in Lebanon that are based on Western concepts of mental disorders, cultural adaptation is needed.

Cultural and contextual adaptation is an important step of formative work in delivering a pre-existing mental health intervention to culturally divergent client groups (Heim *et al*. in press) in potentially diverse settings. Several studies showed that adapted interventions have high effect sizes and that adapting psychological interventions to the local culture can increase their effectiveness (Hall *et al*. 2016). Two meta-analyses found higher effect sizes of adapted interventions than non-adapted ones (Griner & Smith, 2006; Benish *et al*. 2011). One additional meta-synthesis that included studies testing depression treatments in non-Western populations found high effect sizes [standardised mean difference (SMD) of −0.72] for adapted interventions compared with their various control conditions (but not compared with the same unadapted intervention) (Chowdhary *et al*. 2014). Finally, one study compared effect sizes of self-help or minimally guided interventions (i.e. books or online interventions with approximately 15 min of personal guidance per week) that were used cross-culturally. The authors found a high pooled effect size of adapted interventions (SMD  =  0.81) and that the efficacy of the intervention increased with a point increase in the *extent* of cultural adaptation (Harper Shehadeh *et al*. 2016) showing a dose–response effect. On the other hand, in a recent meta-analysis of studies testing psychological interventions for the treatment of depression in low- and middle-income countries, cultural adaption did not affect effect sizes (Cuijpers *et al*. 2018). To what extent – and what kind of – cultural adaptation is important is still subject of current research.

One shortcoming in cultural adaptation research is that adaptation methods are not often described in detail by authors, with two of the above meta-analyses citing incompleteness (within publications) or difficulty in attaining information on cultural adaptation methods (Chowdhary *et al*. 2014; Harper Shehadeh *et al*. 2016). This highlights a need for research teams or intervention implementers to publish their methods of adaptation and report adaptations in a concise way.

Some common elements of methods used across studies that cite adaptation methods include ensuring that cultural adaptation should be ecologically valid and informed by research with stakeholders, use flexible methods with the aim of improving engagement, be acceptable and relevant while maintaining a balance of fidelity to the original intervention, and have a cultural fit (Saez-Santiago *et al*. 2017). It is important to document adaptations as well as the reasons for them (Bernal & Rodriguez, 2012). For systematic documentation and dissemination of methods, it is necessary to conceptualise and report the adaptations that have been made, which can be complex when considering the abstract nature of cultural needs and specificities.

The aim of this article is to share experiences and methods that were used during the contextual adaptation of the Step-by-Step e-mental health behavioural intervention. Hwang (2006) stresses the importance of using bottom-up processes in cultural adaptation, i.e. involving community members and key informants when developing culture-specific interventions, as opposed to top-down cultural adaptations of an existing unadapted treatment developed for other groups. This view parallels the distinction between *etic* and *emic* research in mental health. The *etic* perspective assumes that diagnostic categories of mental disorders and corresponding treatments are universally applicable, whereas *emic* approaches address *cultural concepts of distress* and recommend using cultural-specific treatments and outcome measures (Kohrt *et al*. 2014). We opted for a careful balance between these two approaches: Step-by-Step is based on evidence-based techniques and designed by the WHO, thus developed primarily top-down, but the process of cultural adaptation was based on a bottom-up approach, as described below.

## Methods

### Content preparation

The content of Step-by-Step was first written in simple English to maximise its adaptability and to ensure that people with primary school level education would be able to access the content. This was then translated into classical Arabic by a professional translator. Given the intervention is a narrative story, to make the intervention more engaging relatable, the project partners decided to translate the story into a spoken dialect of Arabic. The translation brief was that it would be representative of the three main dialectic groups in Lebanon: Lebanese, Syrian and Palestinian. The dialect translation was carried out from the classical Arabic translation by the project coordinator (JAR) with the assistance of other local staff (EZ and WK). This translation was then reviewed by one Palestinian, Lebanese and Syrian acquaintance (lay persons) of the project staff who were not familiar with the project. Small changes were made post-review, and then reviewed by a mental health professional to ensure that the original meaning and therapeutic aspect had not been lost throughout the adaptation.

### Selection of participants

**Fig. 1.**
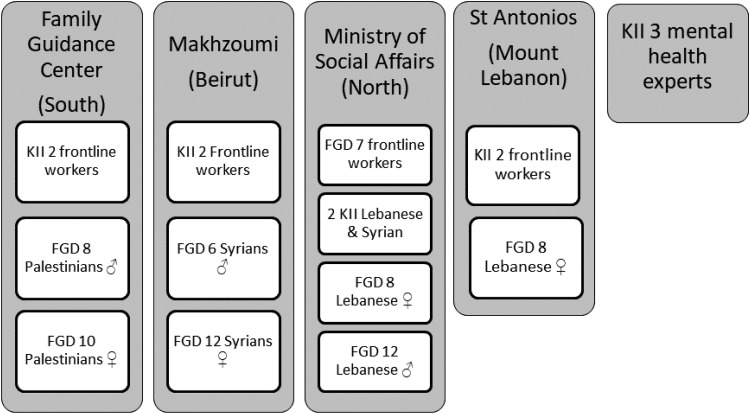
Diagram showing the distribution and number of key informant interviews (KIIs) and Focus Group Discussions (FDGs) across primary health care centres (PHCCs) (NB: all KIIs and FDGs took place in PHCCs except for the KIIs with mental health experts that were held in private clinic settings).

Participants were first approached by the front-line workers in the primary health care centres (PHCCs) and they provided their consent to be contacted by the research team. The qualitative work consisted of 11 key informant interviews (KIIs) and eight focus group discussions (FGDs) with several target groups recruited through four PHCCs across Lebanon so as to represent different cultures and population groups. Stakeholders ranged from mental health professionals, PHCCs managers and front-line workers who could provide insight based on their familiarity with community presentations of and beliefs around depression and community health-seeking behaviours. Moreover, people from the community (Syrian, Palestinian and Lebanese men and women) took part in the cognitive interviewing as well as detailed in Fig. 1. Table 1 in Appendix 1 details the recruitment of participants.

### Data collection

Most of the data collection was undertaken in January 2017 by the project coordinator (JAR) along with two research assistants that were independent from the research project. The project coordinator contacted participants by phone and took oral consent for participation and scheduled the date for the interview or FGD. The FGDs and KIIs for testing Step-by-Step content were semi-structured and their approach borrowed from the ‘verbal probing’ technique used in cognitive interviewing (Drennan, 2003; Willis & Artino, 2013). Cognitive interviewing is a technique predominantly used for questionnaire design that asks respondents to think out loud as they go through a survey or questionnaire while using probing techniques with the aim of improving the clarity and utility of questionnaire items and ensuring they fulfil their purpose. KIIs lasted 1 h and a half while the FGDs lasted 2 h on average. A sample of the interview guide and adaptation form can be found in Appendix 2.

**Fig. 2.**
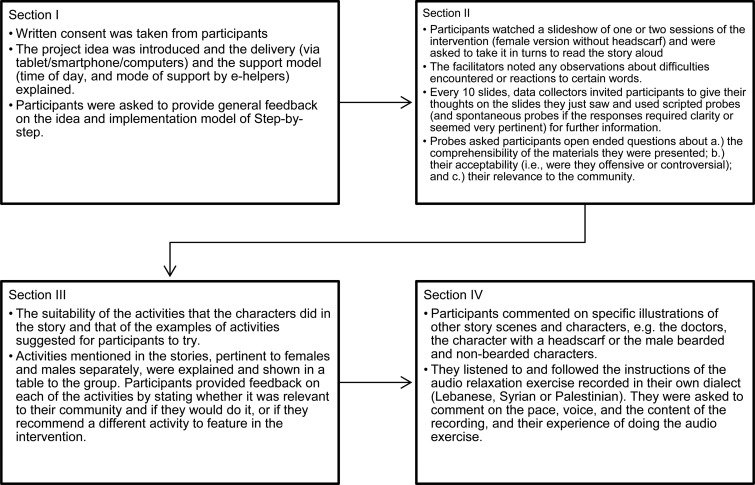
Chart showing the steps and content of the different sections of the cognitive interviewing.

The cognitive interviewing sessions aimed at testing the story content (the illustrations and story were shown on a screen while research assistants read through the story text), audio relaxation exercises (which were played through speakers), and general acceptability and feasibility of the intervention (via discussion). Hence, sessions were divided into four sections: Introduction, idea of Step-by-Step and implementation; Step-by-Step story; Behavioural activation activities; other illustrations and audio. The four sections are detailed in Fig. 2. The KIIs and FGDs adopted the same structure below; however, KIIs with community members (post-FGDs) were more focused on specific sections or questions to clarify any controversial or unclear feedback that was given in the FGDs

### Data analysis

JAR and a research assistant took detailed notes during each session. Suggestions for changing the intervention were recorded by the data collectors on a tracking sheet (original text, proposed change and its justification). Notes were translated into English and certain expressions used in Arabic were kept as they are alongside their corresponding English translation. Within each section of the KII or FGD, information was grouped into themes and subthemes that portrayed similarities and disagreements in opinions in a thematic analysis.

The results of the KIIs and FGDs were organized into tables by JAR and cross-checked by the other two data collection assistants. The results were presented in a 2-day adaptation workshop, attended by nine representatives of the project partners. This included local and headquarters WHO staff, National Mental Health Programme staff from the MoPH, experienced NGO representatives and a local mental health and psychosocial care consultant (who were either internationals or Lebanese nationals). In this workshop, the final decisions as to what content to adapt were made and documented and were informed by the feedback collected from the cognitive interviewing. The team took into consideration feasibility, cost and relevance when deciding on each change recommended, and after in-depth discussions, decisions were made following the majority votes. Following the workshop, amendments to the English and Arabic content and to research procedures were made and reviewed by several team members.

## Results

Results have been arranged according to the themes emerged in the four sections of cognitive interviews (introduction, idea and implementation, Step-by-Step story, behavioural activation activities, illustrations and audios).

### Receptivity/relevance of Step-by-Step

#### Relevance

Consensus was reached among all types of participants across genders and ethnicities, that this intervention is relevant, beneficial and essential to this society because of many prevalent socio-economic and political factors (financial problems, unemployment, marital problems, health issues, war, etc.). Step-by-Step was observed as a relaxing self-care tool, an empowering and educative platform, and the MoPH as a necessary and credible source of information.

#### Receptivity

Most participants stated that they would use the intervention because it (i) reduces the financial and physical barriers to care, (ii) reduces the stigma attached to the use of conventional care, (iii) makes them curious to know what happens next in the story, (iv) would teach them new techniques:

*We were interested in the session, we learned something new. When we start, we will be curious to continue, just like when you are watching a video, you want to watch it all and know how you will benefit*. (Syrian Woman)

#### Non-receptivity

Some of the men across Lebanese, Syrian and Palestinian groups, 2/3 experts and 9/13 front-line workers were concerned that people would not use the intervention because (i) of the lack of awareness of the seriousness of mental distress, (ii) lack of awareness of the benefits of interventions that do not offer financial or material support, and (iii) because they have competing priorities, e.g. their basic survival needs, or are very busy.

[*I suggest to*] *Provide awareness* [*about Step-by-Step*], *and mention the word ‘medical intervention’ to attract people*. (Front-line Worker)

#### Understandability and perception of depression

Participants’ responses on what constitutes depression varied significantly. Some mentioned that depression is an illness, while others referred to it as a simple feeling of unease and even a personal decision. To some, it could be inherited, or caused by external factors and specific problems in a person's life like the wife's behaviour, or problems at work or home. Stigma around mental health was common. One statement was noted:

*When you hear the word psychiatrist, it is associated with craziness or something.* (Syrian Man)

### Content

In this section, participants were asked to discuss the relevance, acceptability and clarity of the content, i.e. the storyline, characters, symptoms and presentation of distress, activity examples, illustrations and audio content.

#### Storyline

Most participants generally accepted the storyline; however, most recommended to emphasise solutions and write them into structured points. Some experts recommended to have an outline in the beginning of each session and add subsections and titles to make the sessions more structured and organised.

Clarity and the level of understanding of the content were assessed by asking users to summarize the slides regularly. Consequently, many misconceptions were depicted, for instance, some were concerned that having a headache or shoulder pain meant that they had depression, while others thought that the hot drinks mentioned in the story such as coffee or tea were a treatment for depression. A major observation was that most people, even the more literate ones, were not at ease with reading the story, hence, they proposed to have an option of recording the story as a video to watch so that it captures people's attention and is easier to follow.

#### Characters

The profile of the character in the version of the story that was shown in cognitive interviewing portrayed a homemaker with children and house duties. She was relatable to some women, but not to working women or people with no spouse or children, who felt that they were not the target of this intervention. Some even warned that the extra focus on children in the story may revive feelings of sadness among the mothers who lost their children back in the war in Syria.

The use of a doctor as the wise character who provides the advice or suggestions for improving one's mood was widely accepted, as the doctor is a very respected figure in the cultures approached and as a general health professional is not stigmatising. They stated that the doctor should be prescriptive and direct in the advice and suggestions s/he makes for getting better. By contrast, the companion should not be prescriptive, but instead provide only the story of how they used instructions that the doctor gave and to encourage the user to do the same thing.

With regard to disclosure about one's feelings, participants reported that spouses did not talk to each other about their feelings, rather they resorted to their best friends or siblings first, because of lack of understanding, prejudice or fear of abandonment (particularly in the case of Syrian women) or fear of showing weakness (mainly men).

*If I tell my husband I'm depressed, he might think I'm of no use and go marry someone else.* (Syrian, Female)

#### Symptoms and presentation of distress

The story does not use medicalising or clinical terms such as ‘depression’ or ‘mental illness’, but rather words like ‘distress’. The participants accepted the use of the local idiom of distress ‘نفسيتي تعبانة’, or ‘tired psyche’ in English, which the team had used in the translated version; however, the symptoms of the mood problem needed revision. The first, unadapted version of the story used the term ‘tired cycle’ to explain the vicious cycle between low mood, avolition and inactivity, which leads to even lower mood. However, FGDs showed that continuous lethargy, increased sleep and crying were not deemed relevant to men, who considered them to be exaggerated and indicative of total hopelessness. Participants in general noted that depressed people in Lebanon maintained their necessary activities and still went to work, yet, they were described to become irritable, tired, sad, frustrated or angry while continuing to function.

*This is hopelessness. But humans shouldn't reach this level of hopelessness. It is an extreme case.* (Palestinian Male)

*People in Lebanon keep doing everything but they feel very sad and angry; so they need to stop and relax instead of staying active.* (Expert)

They identified that people with mood problems experienced lifestyle change in that they stopped doing enjoyable activities and withdrew from their surroundings and social situations. This impression was supported by the experts who thought the story was unrepresentative of many presentations in Lebanon and should be toned down to respond to a wider scope of mild-to-moderate depression or higher functioning.

*In Lebanon, people tend to have severe anxiety and usually say: I'm angry, I'm furious, I'm sad, I'm bored, I'm scared rather than I'm helpless, I'm tired, I'm miserable; they keep active but are anxious.* (Expert)

Additional recommendations about the context and local understanding of mood problems were raised by the participants of the different nationalities. They suggested to add financial problems, stressful work conditions and violence as prominent community problems that might lead to people being depressed.

Recommendations were also given at the level of the text whereby participants suggested different wording translations and metaphors that would appeal better and depict distress and recovery. For instance, the translation of mood in Arabic has some negative connotations, ‘psyche’ would be a better fit. Also, ‘I could feel the sunshine again’ was not commonly said in Lebanon, so was replaced by ‘I felt lightness again’; the term ‘satisfaction’ in Arabic had sexual connotations to some religious groups so it was replaced by ‘feeling of joy or peace’.

#### Illustrations and audio exercises

**Fig. 3.**
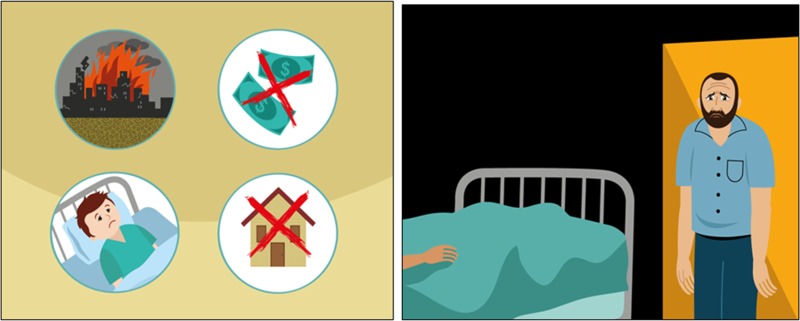
Example illustrations of distressing situations. (Images reproduced with permission from WHO.)

The study team was very aware of the heterogeneity of populations across Lebanon and globally who may use this intervention (e.g. nationals and refugees, high or low social economic status). In the illustrations brief, it was important to specify that illustrations should not include cues targeting one group over another as possible. For example, for depicting adversity and distress, the distressing situation is left interpretable (see Fig. 3).

All participants accepted the illustrations but suggested amendments to the facial expressions of the characters in the story to make them more realistic.

**Fig. 4.**
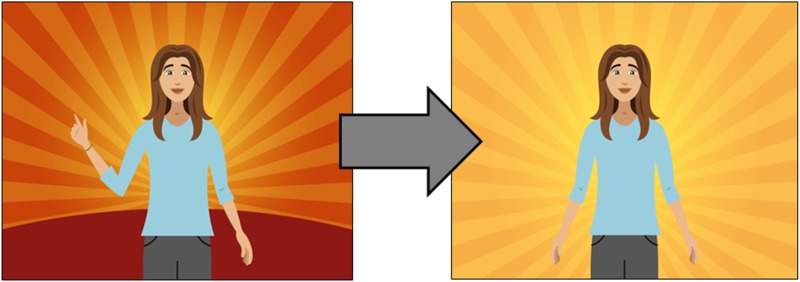
Example of how the colours and hand gesture were changed after adaptation. (Images reproduced with permission from WHO.)

One comment concerned a particular hand gesture of the main character, along with the background colour (bright orange). Participants stated that the gesture in combination with the colour referred to a certain political sign and colour used by a Lebanese political party (see Fig. 4).

As for the audio exercises, people liked them and felt they were relaxing.

*The audio is calming and gives people oxygen.* (Lebanese men)

#### Activities

As part of the behavioural activation component of the intervention, activities are suggested with the main character in the story carrying out activities that will improve his or her mood. Gender specialists at the WHO reviewed the story and highlighted areas where activities were particularly gendered, e.g. women only playing with children, males being angry and women crying, illustrating women in a submissive position compared with the husband, having the male character go out to play backgammon and the woman be indoors all the time. Some changes were made in reaction to this review (e.g. husband helping with preparing food or also playing with children, or both stories referring to anger and crying) and the team tried to have the same activity for both versions of the story wherever possible. However, the local team and participants of FGDs highlighted that gendered activities listed above were the reality in Lebanon and that the story should remain realistic to local norms for people to be able to relate to it. The final story was seen to broadly achieve a balance between removing unhelpful gender stereotypes while remaining realistic to the local situation.

**Fig. 5.**
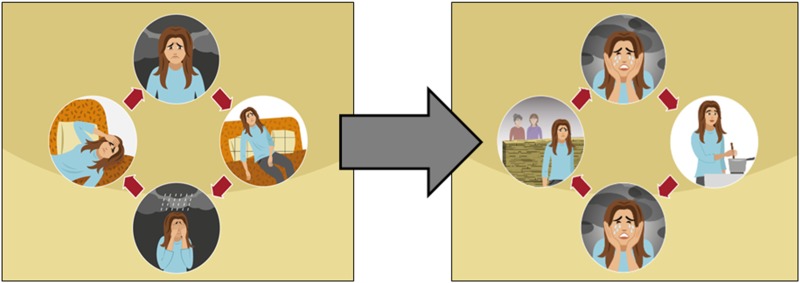
Example of how the ‘tired cycle’ was changed to the ‘sadness and withdrawal cycle’ after adaptation. The new version shows the main character not in a state of inactivity, but continuing with normal activities but in a depressed mood. (Images reproduced with permission from WHO.)

Community participants, as well as health professionals recommended to start with the enjoyable self-care activities first, before doing necessary life activities, which are sometimes a stress factor for people in their community. They also highlighted the fact that people living in Lebanon needed to stop and pause for a while, and think about themselves. Most participants embraced the idea of encouraging people to gain social support and take some rest without feeling guilty about it as being very busy was very often discussed (see Fig. 5).

*It's good to encourage women to gain support from their mothers/relatives and take some rest without feeling guilty.* (Expert)

Participants reviewed the story and a table of activities and commented on each of them. Strong opinions were held in support or against certain activities. An important point was highlighted by the Syrian women and men, who clarified that most Syrian women did not enjoy a high level of independence to go out of their houses alone, thus the example of the woman going out for a walk alone was not perceived as feasible to them. Participants suggested simple indoor self-care activities in sessions 1 and 2, like changing one's appearance and clothes from time to time, personal hygiene, praying, gardening for both genders, reorganizing or tidying the house for women, playing football or exercising for men. Outdoor more complex activities suggestions included going out with friends, spending time in nature, shopping, exercising, fishing, swimming, organizing a family trip, etc. A renowned topic for addition in all FGDs was that religion was an important component in people's lives and could be added as a solution to help against distress. Table 2 in Appendix 3 has examples of how activities were changed after cognitive interviewing.

*You should stress a lot on religion and advice people to listen to prayers, talk about the importance of faith across all religions, faith gives strength.* (Lebanese Women)

The examples below show how illustrations were changed based on the feedback generated, whereas other aspects of the intervention that were changed, are shown in Table 3 in Appendix 4.

### Implementation of Step-by-Step

In this section, participants were asked to discuss the feasibility of Step-by-Step delivered via technology; thus, the feasibility, the length of sessions, the pace of the intervention, the setting preferences, privacy concerns and the support methods were discussed.

#### Feasibility of technologized intervention

Despite the perceived relevance of the intervention, the feasibility of delivering Step-by-Step via e-platform was debatable. When asked whether it would be practical to use the intervention, answers varied among the different groups. Participants across different ethnic groups, mostly Palestinians and Lebanese, a minority among Syrians and 4/13 of the front-line workers believed that the intervention was feasible for many reasons, among which, user friendliness seemed to be an important factor. Also, the wide availability of mobile phones and Internet coverage in all households, even the most vulnerable ones, and the time and place flexibility associated with it, were considered as encouraging factors. Nevertheless, all Lebanese men noted that despite the friendliness of such an intervention and its flexibility, the slow and low quality of Internet connection might be a huge challenge for usage of such an intervention, whereas the majority of Lebanese women were more concerned about their very busy lifestyle and the length of sessions. A big proportion of the Syrian women and men had different concerns though; they mentioned that the main barrier for using an e-intervention was their illiteracy or electronic device illiteracy (especially older adults), and the majority of Syrian women stressed the fact that they did not have access to a phone most of the time as it is shared with their husband. These factors were also foreseen by the majority of the front-line workers.

*if the internet speed is low, we would get angry and delete the app.* (Lebanese, Male)

#### Length of sessions

With few exceptions, most participants had the impression that the sessions were too long and repetitive, and that there was more focus on symptoms and unnecessary details of the story rather than on solutions.

*I prefer the session to last for 5 mins or 10 mins, not more than that.* (Syrian, Male)

#### Pace

Some participants expressed that users might need more than a week to transition from one session to another and might need reminders to complete the sessions.

*It's very difficult to get out of the sad phase in 1 or 2 weeks, this needs more time.* (Syrian, Female)

#### Setting preferences

Preferred place of usage (e.g. at home, in public place) and time (e.g. after work) depended on gender and whether participants were homeworkers or worked outside. Most participants preferred to use Step-by-Step on their own phones instead of using tablets provided in the PHCCs for convenience and privacy and due to lack of time.

*I don't have time to come to the center to user it, especially that I can use it on my phone at home, on my convenience.* (Lebanese, Female)

#### Accessibility, privacy and security

Lebanese and Palestinian participants reported that they did not have any problem using Step-by-Step and providing their contact information, while Syrian men and women and some Lebanese participants in the North area were more sceptical about it and declared that they might put fake numbers as they feared being spied on by certain agencies. Participants recommended the project to be disseminated through the local PHCCs because they are trusted, and to put the MoPH logo so that it adds credibility to the intervention. This is reflected in the following statement:

*Some people might not like to enter their number, especially that there is an unknown organization involved and our information would be stored there. We would trust the MoPH better.* (Lebanese women in the North)

#### E-helper support

When asked about the e-helper support, participants preferred to get an introductory call instead of an email, to get a notification before the call from the e-helper, and to get the number of the e-helper who would call them in advance. This finding was stressed particularly when phones were shared with spouses (mainly for Syrians). Male and female participants reported being less desirable husbands or wives if their partner should find out they had emotional problems. But trying to keep the identity of the caller from their partner could raise questions about their fidelity and even put them at risk of gender-based violence. Syrians preferred to get phone calls and talk to the e-helper, while some of the Lebanese preferred messages only.

## Discussion

Through carrying out in-depth cognitive interviewing with community members, mental health professionals and front-line primary health care staff, valuable information about the content, implementation and the prospective use of the WHO Step-by-Step programme was gathered and used to adapt the intervention to enhance cultural acceptability among different groups in Lebanon.

The methods used for the adaptation of Step-by-Step are not dissimilar to other methods used for gathering data to inform adaptation of psychological interventions in low- and middle-income countries. In a paper providing an overview of the procedures adopted by three such studies, the wider Step-by-Step project somewhat parallels the methods of these research teams in drawing upon the UK Medical Research Council framework for the design and development of complex interventions (Patel *et al*. 2011; Craig *et al*. 2013). This is by using a multi-phase approach (exploratory or formative work on context, feasibility and piloting with process evaluation before definitive testing), of which this study entails part of the first phase. We have used a systematic and documented approach to gathering data for adapting the intervention, engaging a broad range of stakeholders including health workers and community members and previous to this research phase, a number of Lebanese mental health service providers and public mental health academics for their inputs on intervention development and project planning (Patel *et al*. 2011).

During the adaptation workshop based on the qualitative data, the team made decisions about what characteristics and content to revise while maintaining the fidelity of the intervention. Adaptations led to the intervention being approximately 30% shorter, some illustrations being re-drawn or created, and many other content changes and methodological considerations taken into account. One very important adaptation referred to how symptoms of depression were explained in the intervention. The original term ‘tired cycle’ was changed into ‘sadness and withdrawal cycle’, and the symptoms were changed from avolition and inactivity to maintaining daily activities but with low mood, irritation and anxiety. From our results, we cannot conclude whether this presentation of symptoms corresponds to an emic cultural concept of distress (Kohrt *et al*. 2014). It may also be that due to the low socio-economic status of many people in a middle-income country such as Lebanon, inactivity is not an option because people have to secure their livelihoods. Ethnographic research on cultural concepts of distress would be required to better understand this concept.

Similarly to the content, decisions pertaining to the delivery method of Step-by-Step were made and are detailed in Table 3 of Appendix 4. Given the range of views on acceptability of the support element of Step-by-Step, one main decision was to give the choice to the participants to choose their preferred method of contact (phone, email, chat), their preferred day and time. This is to prevent any breach of privacy, confidentiality and any intrusiveness of the support.

We did not find a preference for traditional methods of healing as some studies suggest: (Karam *et al*. 2006; Gearing *et al*. 2013) among the cognitive interviewing participants though this is likely because we recruited participants through mainstream medical services. However, our findings parallel results from a systematic review which identified a preference for directive and authoritarian approaches to care, mirrored in our focus groups by the preference of a doctor figure who is prescriptive and directive (Gearing *et al*. 2013). Our findings also dovetail with the barriers identified in this systematic review, such as lack of awareness around mental health, gender issues and stigmatization of people with mental health problems.

### Strengths and limitations

Using community members from the cultural group 1 is adapting to, as was done for this adaptation, is said to be the best way to arrive at the most contextually appropriate revisions of psychological interventions (Hall *et al*. 2016). A recent and large meta-analysis found that most cultural adaptation methodologies take a top-down methodology to adapt interventions, with just four out of 78 studies adapted interventions using a bottom-up, or community-driven approach (Hall *et al*. 2016). A strength of the present study is that it adds to the literature in a context of a lack of published accounts of bottom-up cultural adaptation practices in the region. Furthermore, we ensured that the three main cultural groups in Lebanon were involved, that is Lebanese, Syrian and Palestinian men and women.

This cognitive interviewing had several limitations. First, we recruited participants through primary health care services, which may have biased our results towards opinions that are compatible with medical health care seeking (and therefore not including people more inclined to seek traditional forms of care). Second, the final decision makers as to adaptations did not include Palestinians nor Syrians. Third, though we did engage target community members, for ethical reasons, we did not use target community members who had common mental disorders, so we could have gone one step further with our ecological validity. We did however try to include the local mental health perspective by getting feedback from local mental health experts and from PHCC staff.

### Implications and future research

In light of limited evidence on methods of cultural adaptation in current literature, this paper provides a detailed report on methods and results of a bottom-up, cultural adaptation process of an evidence-based online intervention for the treatment of depressive symptoms in Lebanon.

Hopefully, this will encourage other researchers and implementers to use bottom-up approaches to adaptation and report on their experiences. So far, evidence on the importance of cultural adaptation is mixed, with some meta-analyses showing a higher efficacy of adapted interventions compared with non-adapted ones (Benish *et al*. 2011; Hall *et al*. 2016), whereas one more recent meta-analysis found no effect (Cuijpers *et al*. 2018). Future research will show whether cultural adaptation such as implemented in the present study increases the usability and effectiveness of interventions. But regardless of these very important aspects, we are convinced that using culturally specific illustrations and examples, and making sure the content is meaningful and non-offensive, should be a minimal standard when delivering interventions to culturally diverse groups.

## Conclusion

Bottom-up cognitive interviewing with community members, front-line health workers and mental health professionals provided very relevant information which will hopefully contribute to increase the relevance and acceptability of the Step-by-Step intervention. It was important to have community members involved in informing decision-making and have experts ensure that final decisions of what changes to make to the intervention would maintain fidelity of the intervention post-adaptation.

## Appendix 1

**Table A1.** Shows the steps and criteria for participant selection for the cognitive interviewing.

Recruitment of participants	Centres were selected based on the availability of mental health services, on their participation in the National Mental Health Programme activities, and on typical patients’ demographics (Lebanese, or Syrian or Palestinian nationalities)
	In total, three mental health professionals and 13 PHCC front-line workers were interviewed, 64 community members including Lebanese, Syrian and Palestinian nationalities participated in FGDs, and two community members were interviewed individually for more in-depth information
	PHCCS helped recruit participants from among their beneficiaries for the FGDs by approaching them while in the waiting room. Two front-line workers were interviewed from each centre and one or two FGDs took place in each centre for women (*n* = 38) and men separately (*n* = 26)
	People of these three nationalities, above 18 years of age, and with primary school level literacy (in order to be able to engage with the Step-by-Step materials) were included in the study
	The focus group participants were not screened for depression. Participants completed a written consent form with the assistance of the data collector where necessary and were remunerated US$15 for their time

## Appendix 2

Interview questions and example adaptation monitoring form – Mental Health Experts/Front-line personnel

Date of interview:

Population with which the interviewee works: (Lebanese, Palestinian, Syrian)

Area of Lebanon in which the interviewee works:

Year of experience in mental health:

Sex of interviewee:

Thank you for being willing to provide feedback on this e-Mental Health intervention for populations living in adversity. We will be showing you segments of the intervention. At various points, we will stop to ask you questions about its relevance and acceptability. If you notice something prior to the point at which we stop, please call out and we will stop immediately.

As you listen and read, please ask yourself the following questions:

- Does the content make sense for – or is it relevant to – Lebanese nationals and Syrian or Palestinian refugees?
- Is the content appropriate for the target population? i.e., is it meaningful, understandable and not offensive or upsetting?
- How could we change the content or the visuals or any other aspects of the intervention to make it more relevant and acceptable?

Please keep in mind that you are seeing one version of the story. There will be several other versions with different characters (show the different characters). Participants can choose which character to watch. The story you will watch is also the female version. There will be a male version with different activities but the same strategies.

Can we begin?

****************************

**Table:**

Stage of adaptation	Original text including document name and page number	Proposed change	Justification to change original text	Notes	Change agreed
Contextual research			□ Not understandable □ Inappropriate □ Irrelevant □ Other		□ Yes □ No
Contextual research			□ Not understandable □ Inappropriate □ Irrelevant □ Other		□ Yes □ No

## Appendix 3

**Table A2.** Shows some examples of original story activity suggestion alongside the suggestions from participants to make amendments.

Original activity or activity suggestion	Participant feedback and suggestion for amendment	Decision made during the adaptation workshop
Making and drinking a cup of tea (Both genders)	Drinking tea was a simple, pleasant and common activity, yet, to be replaced by ‘drinking a hot drink’ which could be coffee, tea or matté in the case of a certain population in Lebanon, to avoid misleading people into believing that tea had herbal or therapeutic effects against depression	Change to drinking a hot drink and reduce the frequency that this activity features in the story
Going for a walk (Both genders)	Walking was deemed to be very beneficial; yet, instead of walking alone, walking with company could be an option for some Syrian women who cannot go outside unaccompanied	Walking was maintained as a very healthy activity. In some of the illustrations, the character is walking with family of friends but text does not specify alone or in company
Reading a book (Both genders)	Reading a book was thought to be too complicated and stressful for depressed people. Also, it does not seem to be very common among adults and might be offensive to people with low literacy	This activity was removed
Playing with one's children (Both genders)	Playing with one's children was thought to be a stressful activity that adds burden to a depressed person. It was advised by the participants and the experts to postpone this activity in the story from session 1 to session 4 or 5	Overall, the references to children were reduced, men were included in illustrations with the children and this activity was included for both men and women
Listening to favourite singer (Both genders)	Listening to favourite singer was criticized by some religious people who noted that listening to music was prohibited in their religion. Participants suggested that it could be kept as an option ‘listen to music’ and music could be classical	This was changed to listening to favourite music
Going to the market first alone then with the children (Both genders)	Taking the kids to the market could be very stressful and difficult to a depressed person and even embarrassing in case that person is from a low socio-economic class. The suggestion was to replace it with going to the market alone and remove going with children or postpone it to session 4 or 5	This was changed to taking the family out for the day, with a practice outing of getting picnic supplies from the market alone
Inviting, preparing and hosting a family dinner (Females only)	Invite family to come over for dinner is good and enjoyable but costly and impractical for some Syrians since several families live in the same apartment. Thus, making dinner and or dessert could be a better alternative	This was maintained for women as a complex social activity but with four extra people (rather than saying ‘extended family’)
Going to meet friends to play chess at a café (Males only)	Meeting a friend to play chess in a café was considered relevant and applicable, yet better replace chess with cards or backgammon which are much more common in this culture. Also, encourage men to spend time with their families at home as well	This was replaced with going to the cafe or a friend's house to play backgammon

## Appendix 4

**Table A3.** Shows the changes pertaining to the different aspects of the content and delivery method of the intervention.

Topic	Decisions based on results from FGDs and KIIs
Length of sessions	The project team took on board the advice from many participants of summarising the story into more concise sessions by reducing redundancies and removing unnecessary details of the story (approximately two-thirds of its original length)
Pace of intervention	The time gap between the sessions was extended, users are given 10 days to complete one session before sending a reminder to start the next
Support time and method	Project team took into consideration the time preferences of the community and increased the flexibility of the support methods by providing more options (phone, email or chat support)
Role of characters	Participants suggested that the doctor and narrator should have different roles to add credibility to the advice. Therefore, a prescriptive role was given to the doctor, and narrative role to the main character
Inclusiveness	Based on the comments from single and working female participants who had difficulties in seeing themselves related to the narrator, the project team decided to include less illustrations of and reference to children in the story and is considering making a version of the story for single users
Clarity of the content	The team responded to the comments related to the misconceptions of the therapeutic examples used, and the difficulty of reading the story. The story was made into a video and the psychoeducational parts were reformulated
Concept of depression	A central part of the story, the ‘tired cycle’ was updated to the ‘sadness and withdrawal cycle’ in response to participants’ suggestions. See Fig. 3 for an example of how this element of the story changed
Illustrations	One illustration was changed with regard to colour and hand gesture because the original referred to a political party in Lebanon (see Fig. 4)

## Appendix 5

Focus Group Discussions with Potential Users

Consent form

Ministry of Public Health in Lebanon

My name is -------------------------. I am working with the Ministry of Public Health in Lebanon on a research project conducted by the World Health Organization with the Ministry of Public Health in Lebanon, and supported by a charitable foundation in Switzerland, Fondation d'Harcourt. I am inviting you to participate in a focus group discussion in order to give us in-depth feedback on the content of an internet-based mental health program. This program is called ‘Khoutweh Khoutweh’.

Before we begin, I would like to take a few minutes to explain why I am inviting you to participate and what will be done with the information you provide. Please stop me at any time if you have questions about the focus group. After I've told you a bit more about the project, you can decide whether or not you would like to participate.

‘Khoutweh Khoutweh’ is a minimally guided, electronic self-help program aimed at addressing the mental health needs of Lebanese nationals and Palestinian and Syrian refugees living in situations of adversity in Lebanon and experiencing difficult emotions. This intervention will eventually be used in selected primary health centres in Lebanon. Before using this intervention, we want to make sure that it is relevant, and that the content is easy to understand and acceptable. We are inviting you to participate in a focus group discussion to provide in-depth feedback on whether the content of the intervention is understandable, acceptable, and relevant. You have been selected from the beneficiaries of this primary health centre/non-governmental organization to participate in the research. The interview will take a maximum of 3 hours. Approximately 10 other participants will also be asked to participate.

If you agree to participate, we will show you the content of the intervention and you will be asked to answer several questions along with a group of other participants. Questions will address the relevance and acceptability of the translated and adapted text, audio scripts and illustrations to your culture. Your participation is entirely voluntary and you have the right to withdraw your consent or discontinue participation at any time without penalty. If at any time and for any reason you would prefer not to answer any questions, please feel free to skip those questions. If at any time you would like to stop participating, please tell me. Your decision to withdraw will not involve any penalty or loss of benefits to which you are entitled. Discontinuing participation in no way affects your relationship with your health center, the non-governmental organization or any of the three partners that are implementing this program (Ministry of Public Health, the World Health Organization and Foundation d'Harcourt). No one from *insert name of NGO* will be present when we are conducting the focus group discussion with you.

Your participation in this focus group does not involve any physical risk or emotional risk to you beyond the risks of daily life. There is a slight chance that listening to the story might cause you some emotional discomfort. If this happens, we can stop immediately and you can choose to discontinue the interview or wait a while and then continue.

You receive no direct benefits from participating in this research; yet, you may learn some tips to support your own mental health. Your participation does help to improve the program and inform the adaptation of an intervention that will have potential to promote mental health of persons living in adversity in Lebanon. Refreshments will be available; there are no financial benefits for participating in the discussion but we will provide LL10,000 for any transportation costs you might incur.

To secure the confidentiality of your responses, no identifiers will be kept. We will only note age, gender, nationality, and marital status to use for descriptive statistics. We will take handwritten notes without referring to your identity so that we accurately remember all the information you provide while preparing the project report. Hard copies will be stored in a locked cabinet in the office of the research. Soft copies will be saved on a password protected computer. Data access is limited to the Principal Investigator and staff working directly on this project. Records may be monitored by the IRB, but all information will remain confidential. All data will be destroyed responsibly after the required retention period (usually three years).

You will be provided a copy of the consent form. If you have any questions, feel free to ask them now.

You may also communicate now or in the future with the **Principle Investigator** responsible for the research: **Dr. Rabih El Chammay** Phone: +961 1 611 672 ext.: 125; E-mail: rchammay@moph.gov.lb

*********

Are you interested in participating in the focus group?

o Yes

o No

**Consent to quote from the focus group:**

I may wish to quote from this focus group either in presentations or in reports resulting from this work. A pseudonym will be used in order to protect your identity. You can still participate in the interview even if you do not want us to use quotes.

Do you permit me to quote from this focus group?

o Yes

o No

Participant's name: ____________________________ Date: _____________________

Signature: ____________________________

Signature of person obtaining consent: ________________________________

Printed name of person obtaining consent: _____________________________

Date _____________
